# Consequences of SPAK inactivation on Hyperkalemic Hypertension caused by *WNK1* mutations: evidence for differential roles of WNK1 and WNK4

**DOI:** 10.1038/s41598-018-21405-x

**Published:** 2018-02-19

**Authors:** Chloé Rafael, Christelle Soukaseum, Véronique Baudrie, Perrine Frère, Juliette Hadchouel

**Affiliations:** 1 Institut National de la Santé et de la Recherche Médicale (INSERM), Unit 970, Paris Cardiovascular Research Center, Paris, France; 2 Universités Paris-Descartes et Paris-Diderot, Sorbonne Paris Cité, Paris, France; 3 Sorbonne Université, Paris, France; 4 Assistance Publique - Hôpitaux de Paris (AP-HP), Paris, France; 5 INSERM UMR_S1155, Tenon Hospital, Paris, France; 6 INSERM UMR_S1176, CHU de Bicêtre, Le Kremlin-Bicêtre, France

## Abstract

Mutations of the gene encoding WNK1 [With No lysine (K) kinase 1] or WNK4 cause Familial Hyperkalemic Hypertension (FHHt). Previous studies have shown that the activation of SPAK (Ste20-related Proline/Alanine-rich Kinase) plays a dominant role in the development of FHHt caused by *WNK4* mutations. The implication of SPAK in FHHt caused by *WNK1* mutation has never been investigated. To clarify this issue, we crossed *WNK1*^+*/FHHt*^ mice with SPAK knock-in mice in which the T-loop Thr243 residue was mutated to alanine to prevent activation by WNK kinases. We show that *WNK1*^+*/FHHT*^:*SPAK*^*243A/243A*^ mice display an intermediate phenotype, between that of control and *SPAK*^*243A/243A*^ mice, with normal blood pressure but hypochloremic metabolic alkalosis. NCC abundance and phosphorylation levels also decrease below the wild-type level in the double-mutant mice but remain higher than in *SPAK*^*243A/243A*^ mice. This is different from what was observed in WNK4-FHHt mice in which SPAK inactivation completely restored the phenotype and NCC expression to wild-type levels. Although these results confirm that FHHt caused by *WNK1* mutations is dependent on the activation of SPAK, they suggest that WNK1 and WNK4 play different roles in the distal nephron.

## Introduction

Familial Hyperkaliaemic Hypertension (FHHt), also known as Gordon’s syndrome or Pseudohypoaldosteronism type II (PHAII), is a rare genetic form of hypertension associated with hyperkalemia and metabolic hyperchloremic acidosis (OMIM #145260)^1^. All these symptoms are corrected by thiazide diuretics, which inhibit the activity of the Na^+^-Cl^−^ transporter NCC, encoded by the *SLC12A3* gene^1^. FHHt is caused by mutations in one of at least four genes: *WNK1* [With No lysine (K) 1], *WNK4*, *KLHL3* (Kelch-Like family member 3), and *CUL3* (cullin-3)^2–4^. The associated proteins belong to the same regulatory pathway, as WNK1 and WNK4 are recruited by KLHL3 for ubiquitination by the Cul3-E3 RING-type ubiquitin–ligase complex^5^.

The sensitivity of patients to thiazide diuretics strongly suggested that FHHt is mainly caused by a gain of activity of NCC in the distal nephron. The role of NCC in regulating blood pressure has been established by the discovery of inactivating mutations of the *SLC12A3* gene, which cause Gitelman’s syndrome, an inherited disorder that is the mirror image of FHHt, with arterial hypotension, renal salt wasting and hypokalemic metabolic alkalosis (OMIM #263800)^6,7^.

Therefore, several studies focused on the mechanisms underlying the regulation of NCC by WNK1 and WNK4. *In vitro* studies have demonstrated that the WNKs activate two kinases, SPAK (Ste20-related Proline/Alanine-rich Kinase) and OSR1 (oxidative stress-responsive kinase 1)^8^, which can in turn phosphorylate and activate NCC^9^. SPAK and OSR1 can also activate the Na^+^-K^+^-2Cl^−^ cotransporters, NKCC1 and NKCC2, and inhibit the K^+^-Cl^−^ cotransporters, KCC1–4^10^. *In vivo*, the expression and phosphorylation of NCC is reduced when SPAK is inactivated^11,12^. Finally, the fact that the mutation of a key SPAK/OSR1 phosphorylation site on NCC (Thr60) causes Gitelman’s syndrome confirmed the major role of SPAK/OSR1 in the regulation of NCC activity *in vivo*^13^.

Several studies have then demonstrated that the activation of SPAK, but not OSR1, by WNK4 is a key step in the pathogenesis of FHHt. First, SPAK abundance and phosphorylation are increased in a mouse model of FHHt caused by a missense mutation of *WNK4* (*Wnk4*^*D561/*+^ mice)^14^. Second, the genetic inactivation of SPAK in the same mouse model corrects all FHHt disorders and allows the restoration of NCC abundance and phosphorylation to the wild-type level^15,16^. By opposition, *Wnk4*^*D561/*+^ mice bearing a OSR1 inactivation in the nephron (*Wnk4*^*D561/*+^:*KSP-Osr1*^−/−^) still display a complete FHHt phenotype^16^, even though OSR1 deficiency in the nephron causes hypokalemia under normal diet and systolic hypotension when mice are fed a low Na^+^ diet^17^.

The implication of SPAK in FHHt caused by a mutation of *WNK1* has never been investigated. We previously generated the *WNK1*^+*/FHHt*^ mouse model, carrying a heterozygous deletion of *WNK1* first intron, corresponding to the mutation found in FHHt patients^18^. This deletion leads to an increased expression of L-WNK1, the ubiquitous kinase isoform of WNK1, in the DCT and the CNT. *WNK1*^+*/FHHt*^ mice display hyperkalemia, hypertension and metabolic acidosis as well as increased NCC abundance and phosphorylation. In this model, SPAK abundance and phosphorylation measured by western blot on renal cortex extracts are similar to those of control mice. However, we showed by immunofluorescence that the abundance of total and phosphorylated SPAK increases near the apical membrane of DCT cells in *WNK1*^+*/FHHt*^ mice compared to controls^18^. To investigate whether or not SPAK is necessary to trigger the FHHt caused by *WNK1* mutations, we characterized *WNK1*^+*/FHHt*^ mice in which SPAK activation by WNK kinases is prevented by the mutation of the T-loop Thr243 residue into alanine (*SPAK*^*243A/243A*^ mice)^11^. We show here that *WNK1*^+*/FHHT*^:*SPAK*^*243A/243A*^ mice display an intermediate phenotype, between that of control and *SPAK*^*243A/243A*^ mice. Similarly, NCC abundance and phosphorylation levels decrease below the wild-type level in the double-mutant mice but remain higher than in *SPAK*^*243A/243A*^ mice, which is different from what was observed in *Wnk4*^*D561/*+^:*SPAK*^*243A/243A*^ ^15^. These results suggest that *WNK1* and *WNK4* play different roles in the distal nephron even if in both cases SPAK deficiency is sufficient to preclude the development of FHHt in mice.

## Results

### SPAK inactivation in *WNK1*^+*/FHHt*^ mice results in an intermediate phenotype, between control and SPAK mutant mice

**Figure 1:**
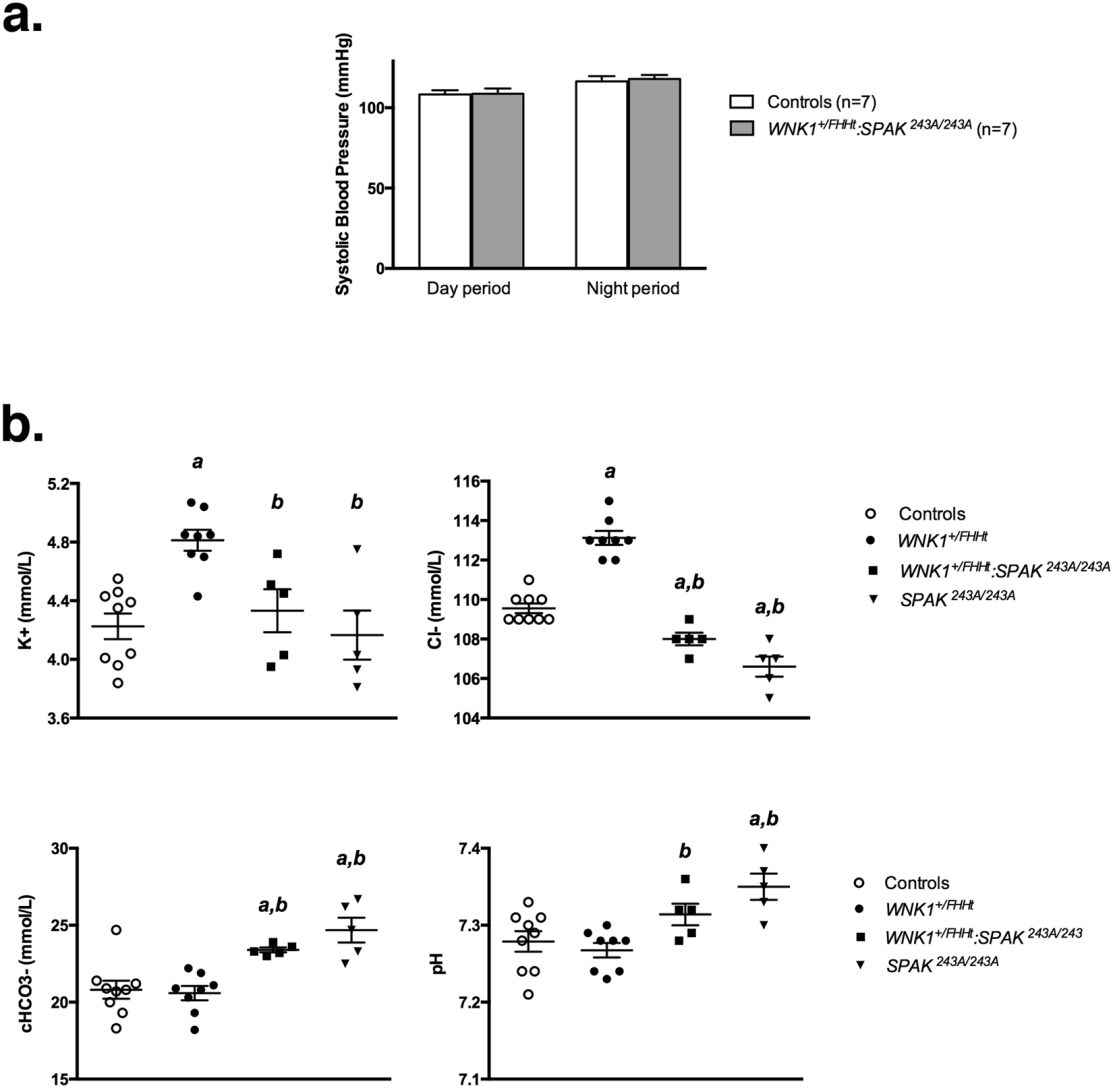
SPAK inactivation in *WNK1*^+*/FHHt*^ mice restores blood pressure but leads to a mild metabolic hypochloremic alkalosis. (**a**) Averaged systolic blood pressure during the 12h-day or -night period measured by radiotelemetry in *WNK1*^+*/FHHt*^:*SPAK*^*243A/243A*^ and control mice. No difference was observed between the two groups of mice (Student’s test). (**b**) Plasma electrolyte concentrations in mice of each genotype at baseline. *SPAK*^*243A/243A*^ mice display a hypochloremic metabolic alkalosis while *WNK1*^+*/FHHt*^ mice exhibit a typical FHHt phenotype. SPAK inactivation in *WNK1*^+*/FHHt*^ mice leads to the development of a hypochloremic metabolic alkalosis, which tends to be less severe than in *SPAK*^*243A/243A*^ mice. Values are means ± s.e.m. ^a^*p* < 0.05 vs. controls. ^b^*p* < 0.05 vs. *WNK1*^+*/FHHt*^ (Kruskal-Wallis one-way analysis of variance followed by Mann-Whitney test). Experiments were performed on 9 control, 8 *WNK1*^+*/FHHt*^, 5 *WNK1*^+*/FHHt*^:*SPAK*^*243A/243A*^ and 5 *SPAK*^*243A/243A*^ male mice.

We previously demonstrated an increased blood pressure in *WNK1*^+*/FHHt*^ compared to control mice (122.9 ± 4.8 mmHg in *WNK1*^+*/FHHt*^ vs 112.0 ± 3.2 mmHg in controls, p = 0.047)^18^. We measured blood pressure in controls and *WNK1*^+*/FHHt*^:*SPAK*^*243A/243A*^ mice by a real-time radiotelemetry approach. *WNK1*^+*/FHHt*^:*SPAK*^*243A/243A*^ mice are normotensive (Fig. 1a) compared to control littermates.

As previously described^18^, *WNK1*^+*/FHHt*^ mice exhibited hyperkalemia and hyperchloremia while *SPAK*^*243A/243A*^ mice exhibited hypochloremic metabolic alkalosis (Fig. 1b). As for blood pressure, the inactivation of SPAK in *WNK1*^+*/FHHt*^ mice restores plasma potassium concentration to normal. Surprisingly, it also results in mild hypochloremic metabolic alkalosis (Fig. 1b). However, plasma chloride concentration tends to be higher in *WNK1*^+*/FHHt*^:*SPAK*^*243A/243A*^ mice than in *SPAK*^*243A/243A*^ mice. In conclusion, the double-mutant mice have an intermediate phenotype, between control and *SPAK*^*243A/243A*^ mice, suggesting that the inactivation of SPAK in *WNK1*^+*/FHHt*^ mice impairs ion transport in the distal nephron.

**Figure 2:**
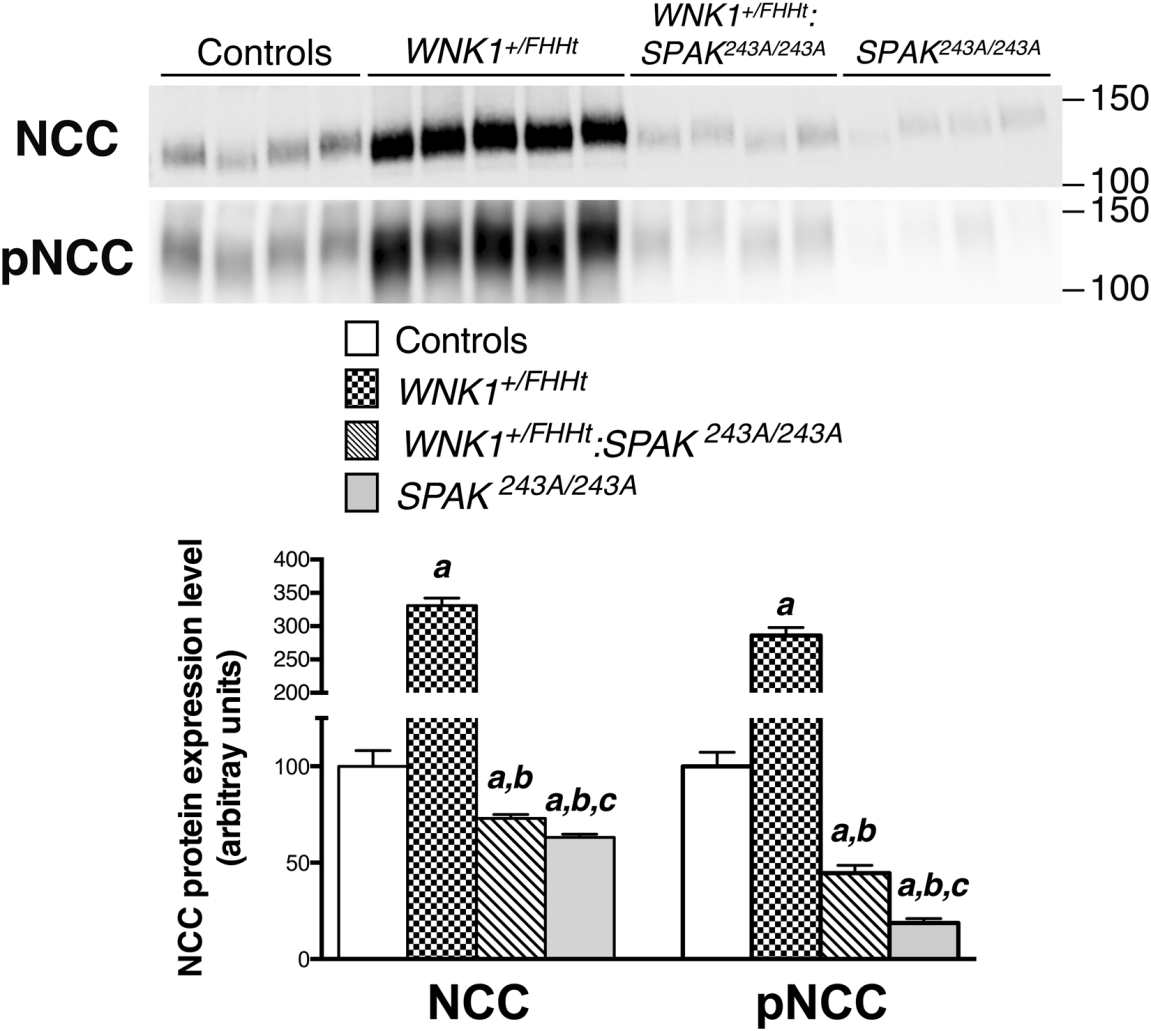
SPAK inactivation in *WNK1*^+*/FHHt*^ mice decreases NCC abundance and phosphorylation below control levels. Upper panel: Representative immunoblots of NCC and phosphorylated NCC-Thr53 performed on the membrane-enriched fractions of the renal cortex of mice of each genotype. Lower panel: Densitometric analysis. NCC abundance and phosphorylation are increased in *WNK1*^+*/FHHt*^ mice compared to control mice. NCC abundance and phosphorylation in *WNK1*^+*/FHHt*^:*SPAK*^*243A/243A*^ and *SPAK*^*242A/243A*^ mice are corrected below the control levels. Number of animals: 4 control, 5 *WNK1*^+*/FHHt*^, 4 *WNK1*^+*/FHHt*^:*SPAK*^*243A*:*243A*^ and 4 *SPAK*^*243A*:*243A*^ male mice. Values are means ± s.e.m. ^a^*p* < 0.05 vs. controls. ^b^*p* < 0.05 vs. *WNK1*^+*/FHHt*^. ^c^*p* < 0.05 vs. *SPAK*^*243A/243A*^*-WNK1*^+*/FHHt FHHt*^ (Kruskal-Wallis one-way analysis of variance followed by Mann-Whitney test). Immunoblot images are cropped images. The full-length images can be found in Supplementary Figure S3.

This intermediate phenotype is also observed for NCC abundance and phosphorylation. As previously reported^11,18^, these parameters significantly increase in *WNK1*^+*/FHHt*^ mice compared to control mice, while they decrease in *SPAK*^*243A/243A*^ mice (Fig. 2). SPAK inactivation in *WNK1*^+*/FHHt*^ mice leads to a significant decreased in NCC abundance and phosphorylation (Fig. 2). Interestingly, these two parameters decrease in *WNK1*^+*/FHHT*^:*SPAK*^*243A/243A*^ mice below the levels observed in control mice (about 25% and 50%, respectively), but remain above the levels observed in *SPAK*^*243A/243A*^ mice (Fig. 2). This is in contrast with what was observed in *Wnk4*^*D561/*+^:*SPAK*^−/−^ mice, in which NCC abundance and phosphorylation levels were comparable to control levels^16^.

**Figure 3:**
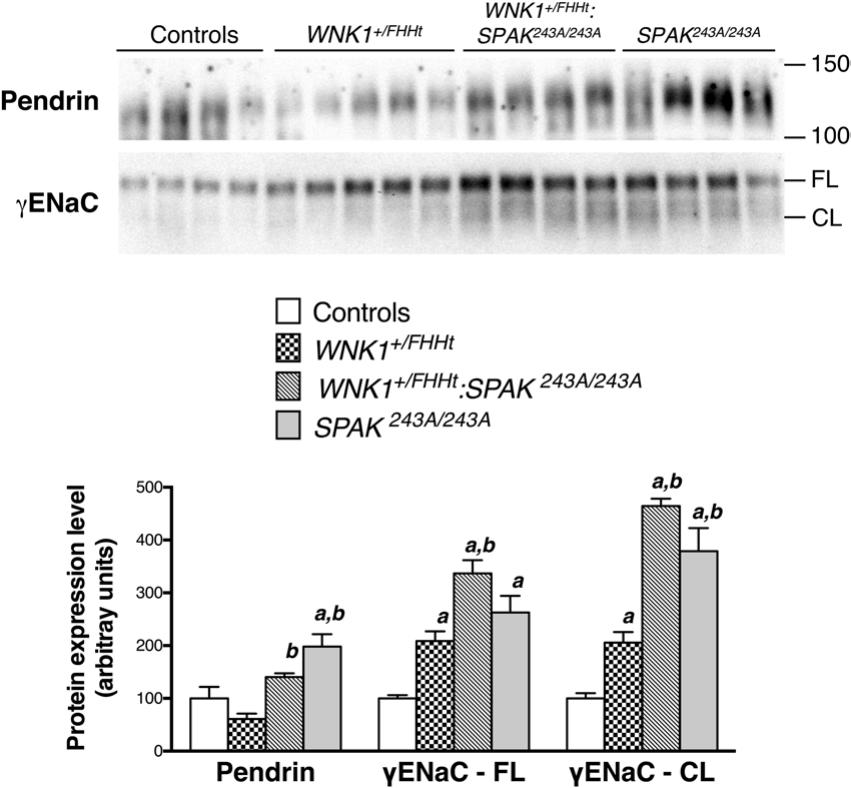
The expression of pendrin and ENaC is increased in the kidney of *WNK1*^+*/FHHT*^:*SPAK*^*243A/243A*^ mice. Upper panel: Representative immunoblots performed with antibodies against the γ subunit of the epithelial channel ENaC and pendrin on the membrane-enriched fractions of the renal cortex of mice of each genotype. FL: Full-length and CL: cleaved form of γENaC, respectively. Lower panel: Densitometric analysis. The abundance and cleavage of γ-ENaC are increased in *WNK1*^+*/FHHT*^:*SPAK*^*243A/243A*^ mice and S*PAK*^*243A/243A*^ mice. The expression of pendrin increases significantly in *WNK1*^+*/FHHT*^:*SPAK*^*243A/243A*^ mice. Number of animals: 4 control, 5 *WNK1*^+*/FHHt*^, 4 *WNK1*^+*/FHHt*^:*SPAK*^*243A*:*243A*^ and 4 *SPAK*^*243A*:*243A*^ male mice. Values are means ± s.e.m. ^a^*p* < 0.05 vs. controls. ^b^*p* < 0.05 vs. *WNK1*^+*/FHHt*^ mice^*FHHt*^ (Kruskal-Wallis one-way analysis of variance followed by Mann-Whitney test). Immunoblot images are cropped images. The full-length images can be found in Supplementary Figure S4.

Grimm *et al*. previously reported that the expression of the sodium channel ENaC and chloride-bicarbonate exchanger pendrin are increased in SPAK knock-out mice^19^. We show here that this is also the case in *SPAK*^*243A/243A*^ mice (Fig. 3). The abundance of pendrin and γ-subunit of ENaC, as well as its cleavage, increase in *WNK1*^+*/FHHt*^:*SPAK*^*243A/243A*^ mice (Fig. 3). The cleaveage of the γ-ENaC subunit is believed to reflect the activation of the channel^20^. The increased pendrin and ENaC abundance probably compensates for the reduced expression and/or phosphorylation of NCC and NKCC2 resulting from the loss of SPAK in *WNK1*^+*/FHHt*^:*SPAK*^*243A/243A*^ mice. Indeed, enhanced expression of pendrin and γENaC has also been reported in *Ncc*^−/−^ mice^21^, probably to compensate for NCC inactivation. The increased ENaC abundance, processing and activity could increase the tubular lumen electronegativity, the driving force for H^+^ secretion, and thus explain the metabolic alkalosis observed in *WNK1*^+*/FHHt*^:*SPAK*^*243A/243A*^ mice. As suggested by Grimm and collaborators, pendrin could be stimulated in response to the alkalosis^19^.

### Expression of OSR1 and Cab39 in *WNK1*^+*/FHHt*^:*SPAK*^*243A/243A*^ mice

The fact that NCC phosphorylation is significantly higher in *WNK1*^+*/FHHt*^:*SPAK*^*243A/243A*^ mice than in *SPAK*^*243A/243A*^ mice strongly suggests that another pathway is activated in the DCT by the overexpressed L-WNK1^18^ to phosphorylate NCC in the absence of SPAK. This hypothesis is indirectly supported by the quantification of the abundance and phosphorylation of NKCC2, another target of SPAK and OSR1. As shown in Supplementary Figure S2. NKCC2 abundance and phosphorylation are decreased in *WNK1*^+*/FHHt*^:*SPAK*^*243A/243A*^ and *SPAK*^*243A/243A*^ mice. However, in contrast to NCC, there is no significant difference between the two groups of mice. This could be due to the lack of activation of an alternative pathway by L-WNK1 since the deletion of intron 1 does not modify the expression and thus activity of L-WNK1 in the cortical ascending limb of the loop of Henle^18^, where NKCC2 is expressed.

**Figure 4:**
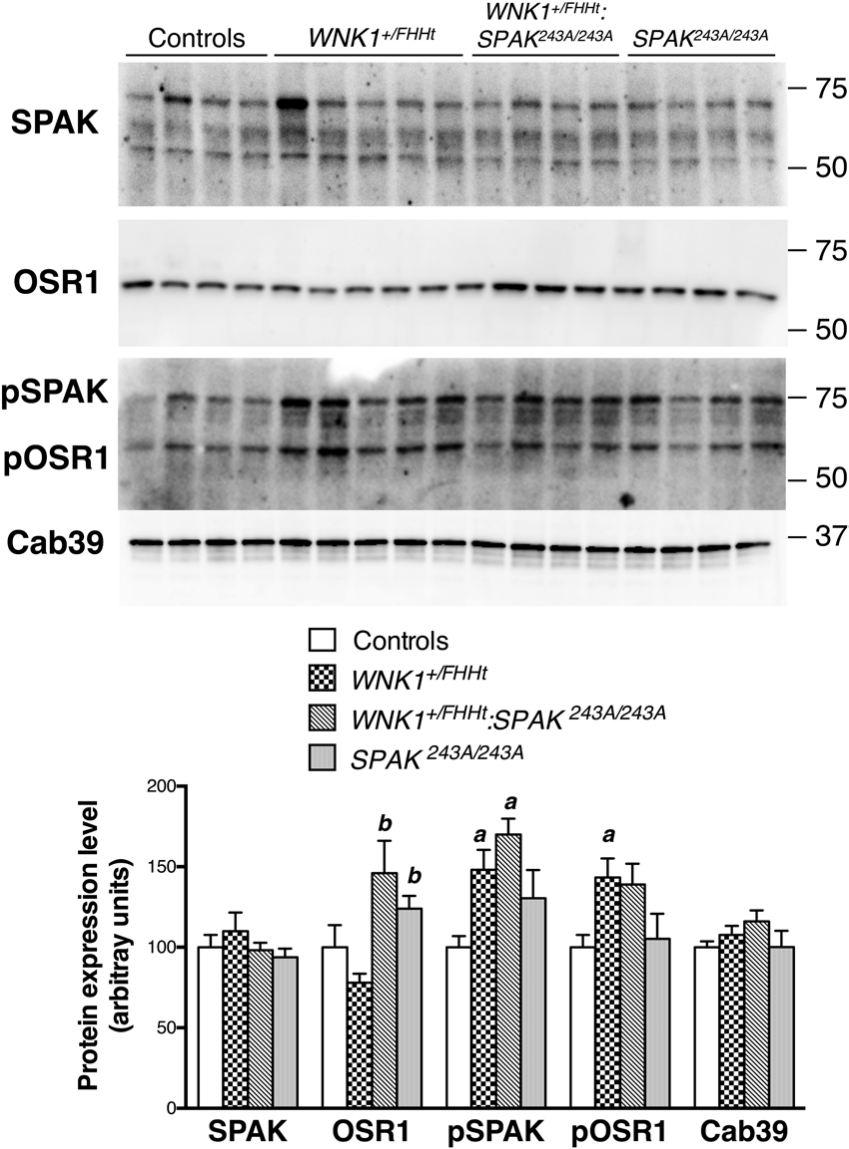
OSR1 remains at the apical membrane of *SPAK*^*243A/243A*^ and *WNK1*^+*/FHHT*^:*SPAK*^*243A/243A*^ DCT, where its phosphorylation tends to increase. Upper panel: Representative Immunoblots performed on total cortex protein extract with antibodies against total SPAK, total OSR1, pSPAK/pOSR1 (S-motif) and Cab39. Lower panel: Densitometric analysis. SPAK inactivation mutation does not affect SPAK expression and S-motif phosphorylation, while it tends to slightly increase OSR1 expression and phosphorylation. Number of animals: 4 control, 5 *WNK1*^+*/FHHt*^, 4 *WNK1*^+*/FHHt*^:*SPAK*^*243A*:*243A*^ and 4 *SPAK*^*243A*:*243A*^ male mice. Values are means ± s.e.m. ^a^*p* < 0.05 vs. controls. ^b^*p* < 0.05 vs. *WNK1*^+*/FHHt*^ mice^*FHHt*^ (Kruskal-Wallis one-way analysis of variance followed by Mann-Whitney test). Immunoblot images are cropped images. The full-length images can be found in Supplementary Figure S5.

In addition to SPAK, two other proteins have been implicated in the regulation of cation-chloride cotransporters: OSR1 and Cab39. The calcium-binding protein Cab39 has been shown to activate NCC in combination with WNK kinases in a SPAK-independent manner^22^. However, Cab39 abundance is similar in all groups of mice (Fig. 4a), suggesting that Cab39 may not be involved in the remaining phosphorylation of NCC observed in *WNK1*^+*/FHHt*^:*SPAK*^*243A/243A*^ kidneys.

We thus hypothesized that OSR1 could be the alternative kinase activated by L-WNK1. L-WNK1 phosphorylates SPAK and OSR1 at two conserved residues, a Thr residue in the T-loop and a Ser residue in the S-motif^8^. While T-loop phosphorylation triggers the activation of SPAK and OSR1, the role of the S-motif phosphorylation is unclear, as its mutation does not affect activation^8^. We could not quantify the phosphorylation of the T loop as it is too low in kidney homogenates^11^. OSR1 abundance is increased in the kidney of *WNK1*^+*/FHHt*^:*SPAK*^*243A/243A*^ and *SPAK*^*243A/243A*^ mice but not of *WNK1*^+*/FHHt*^ mice (Fig. 4). The phosphorylation of the S-motif of OSR1 is increased, albeit not significantly, in *WNK1*^+*/FHHt*^:*SPAK*^*243A/243A*^ mouse kidneys compared to controls (Fig. 4). It also increases in *WNK1*^+*/FHHt*^ but not in *SPAK*^*243A/243A*^ kidneys. The phosphorylation of SPAK S-motif is also increased in *WNK1*^+*/FHHt*^:*SPAK*^*243A/243A*^ mice (Fig. 4). This increase is probably a consequence of the increased L-WNK1 expression^18^.

**Figure 5:**
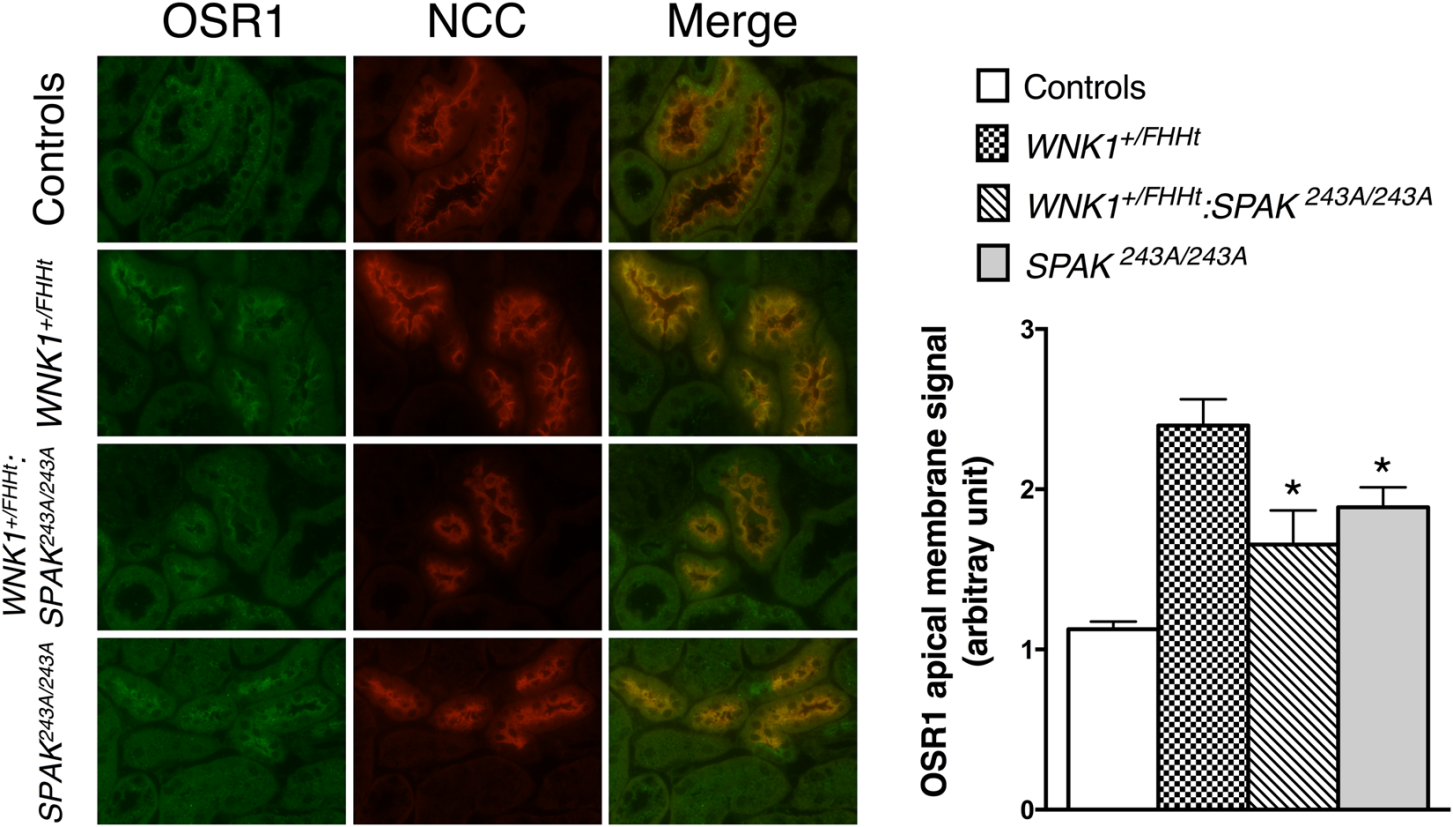
Representative double immunostainingstaining for total OSR1 (in green) and NCC (in red) in the renal cortex of control, *WNK1*^+*/FHHt*^, *WNK1*^+*/FHHt*^:*SPAK*^*243A/243A*^ and *SPAK*^*243A/243A*^ mice. OSR1 colocalize with NCC in distal convoluted tubule cells. Lower panel: quantification of apical OSR1 staining in distal convoluted tubule cells. OSR1 staining is significantly more apical in *WNK1*^+*/FHHt*^:*SPAK*^*243A*:*243A*^ and *SPAK*^*243A*:*243A*^ mice. Number of animals: 4 control, 2 *WNK1*^+*/FHHt*^, 4 *WNK1*^+*/FHHt*^:*SPAK*^*243A*:*243A*^ and 4 *SPAK*^*243A*:*243A*^ male mice. Values are means ± s.e.m. ^a^*p* < 0.05 vs. controls.

We previously showed that SPAK abundance near the apical membrane of DCT cells increases in *WNK1*^+*/FHHt*^ mice^18^, suggesting that the activation of SPAK by WNK kinases stimulates its apical localisation. Similarly, OSR1 phosphorylation and apical localisation increase in mice subjected to potassium depletion^23,24^, a situation in which WNKs are activated^23^. Therefore, we hypothesized that OSR1 localisation near the apical membrane of DCT cells should increase in *WNK1*^+*/FHHt*^:*SPAK*^*243A/243A*^ if OSR1 is indeed stimulated by L-WNK1 to compensate for the absence of functional SPAK. In order to test this hypothesis, we studied OSR1 expression in the DCT by double immunofluorescence staining, with antibodies directed against NCC and OSR1. As shown in Fig. 5, OSR1 is located both in cytoplasmic punctate structures and near the apical membrane in the DCT cells of control mice. In contrast, OSR1 signal is significantly increased near the apical membrane in the DCT of *WNK1*^+*/FHHt*^:*SPAK*^*243A/243A*^ mice, where a stronger colocalisation with NCC can be observed compared to control mice (Fig. 5). In this situation, it is expected that OSR1 could phosphorylate NCC as the phosphorylation of the cotransporter occurs near or within the apical membrane^25^. Taken together, these observations suggest that OSR1 could be activated by L-WNK1 and partially compensate for the lack of SPAK in the DCT *WNK1*^+*/FHHt*^:*SPAK*^*243A/243A*^ mice, in the setting of overexpressed L-WNK1.

Our observations also support the hypothesis that SPAK could be required for OSR1 redistribution to the apical membrane of DCT cells. Indeed, Grimm *et al*. showed that OSR1 is found in dense punctate structures in DCT cells of *SPAK*^−/−^ mice^26^ whereas OSR1 apical localisation is increased in *SPAK*^*243A/243A*^ DCT (Fig. 5).

### Increased WNK1 is localised near the apical membrane of DCT cells

In order to understand how OSR1 can be activated in *WNK1*^+*/FHHt*^:*SPAK*^*243A/243A*^ mice, we characterized the expression of WNK1 and WNK4 in the different groups of mice. While WNK4 expression level is unaffected in *WNK1*^+*/FHHt*^ mice, it significantly decreases in the kidney of *WNK1*^+*/FHHt*^:*SPAK*^*243A/243A*^ and *SPAK*^*243A/243A*^ mice (Supplementary Fig. S2). This decreased expression of WNK4 may result from the DCT hypotrophy caused by the drastic decreased NCC expression, as observed in *Ncc*^−/−^ or *SPAK*^−/−^ mice^26,27^.

**Figure 6:**
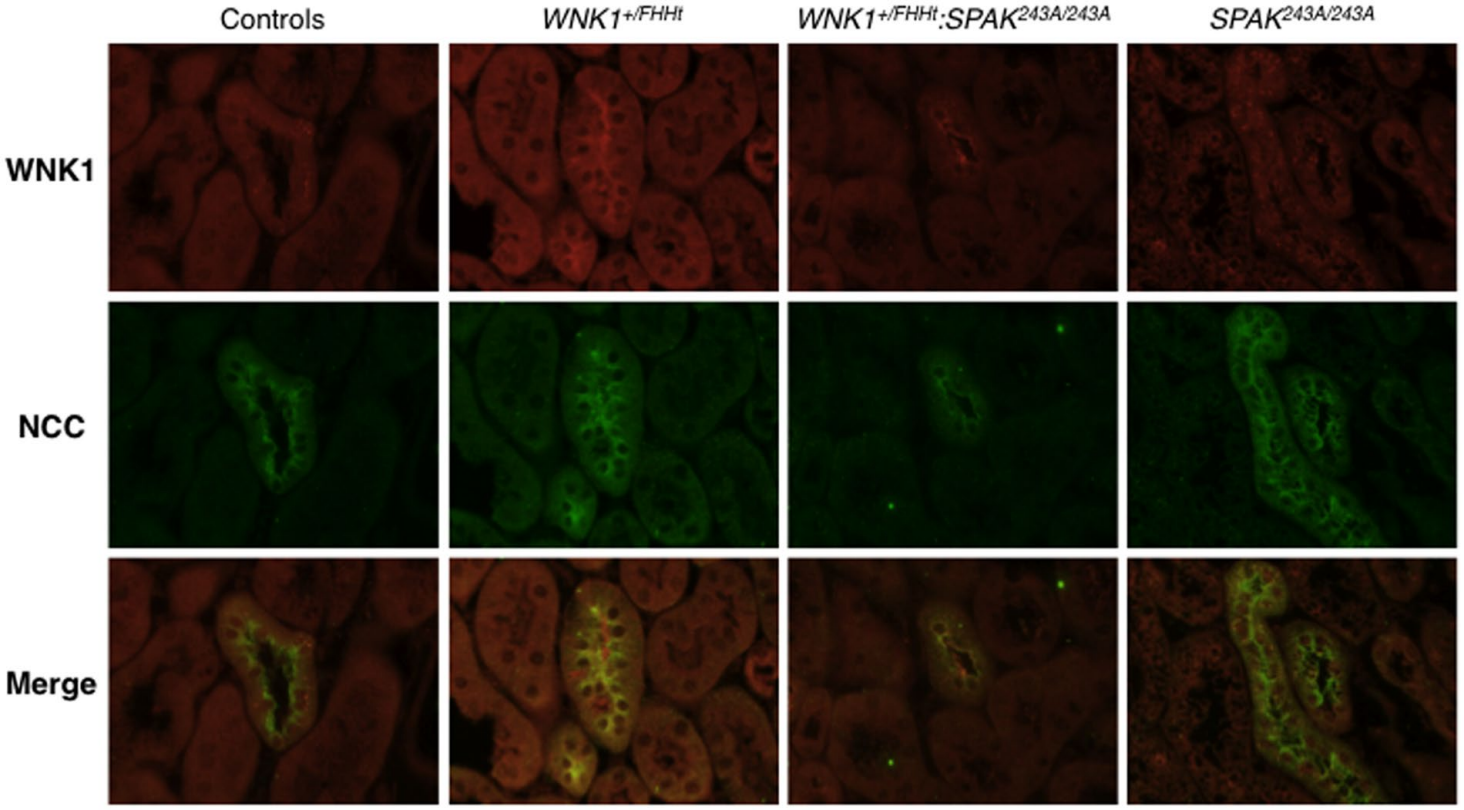
WNK1 cellular localisation is modified when overexpressed. Representative double immunostainingstaining for WNK1 (in red) and NCC (in green) in control, *WNK1*^+*/FHHt*^, *WNK1*^+*/FHHt*^:*SPAK*^*243A/243A*^ and *SPAK*^*243A/243A*^ mice. While WNK1 is located in cytoplasmic puncta in DCT cells of control and *SPAK*^*243A/243A*^ mice, it is observed near the apical membrane of DCT cells in *WNK1*^+*/FHHt*^ and *WNK1*^+*/FHHt*^:*SPAK*^*243A/243A*^ mice, in which L-WNK1 transcription is increased.

We then characterised the expression of WNK1 in the DCT by immunostaining using an antibody directed against a C-terminal fragment of the protein, which thus recognizes both L-WNK1 and KS-WNK1 (Fig. 6). In control and *SPAK*^*243A/243A*^ mice, WNK1 signal is located in cytoplasmic punctate structures in the DCT. In contrast, WNK1 signal is found near the apical membrane in the DCT of *WNK1*^+*/FHHt*^ and *WNK1*^+*/FHHt*^:*SPAK*^*243A/243A*^ mice. In these two groups of mice, L-WNK1 transcription is increased following the deletion of *WNK1* first intron. These observations support our hypothesis that overexpressed L-WNK1 could contribute to a stronger activation of OSR1 and thus NCC in *WNK1*^+*/FHHt*^:*SPAK*^*234A/243A*^ mice compared to *SPAK*^*243A/243A*^.

## Discussion

In this study, we used a mouse model to investigate the consequences of SPAK inactivation in the pathogenesis of FHHt caused by *WNK1* mutation. Indeed, several studies have demonstrated the essential role of SPAK in the pathogenesis of FHHt caused by *WNK4* missense mutations^15,16^ but the implication of SPAK downstream of L-WNK1 in FHHt remained to be formally demonstrated.

We have previously reported the characterisation of *WNK1*^+*/FHHt*^ mice, which we generated by deleting *WNK1* first intron to mimic the large intronic deletions identified in FHHt patients^18^. In these mice, the expression of the ubiquitous catalytic WNK1 isoform (L-WNK1) increases in the DCT and CNT, whereas the expression of the kidney-specific and kinase-defective isoform (KS-WNK1) is unchanged. L-WNK1 overexpression augments the abundance and phosphorylation of NCC, probably via the activation of SPAK. We indeed observed an increased abundance of phosphorylated SPAK near the apical membrane of DCT cells in *WNK1*^+*/FHHt*^ mice^18^. In order to demonstrate the role played by SPAK in the development of FHHt caused by *WNK1* mutation, we crossed *WNK1*^+*/FHHt*^ mice with *SPAK*^*243A/243A*^ mice. The latter carry a Thr243Ala mutation, which precludes the phosphorylation of SPAK T-loop by WNK kinases^11^. *WNK1*^+*/FHHT*^:*SPAK*^*243A/243A*^ mice exhibit normal blood pressure and plasma K^+^ concentration compared to control mice. However, they develop a hypochloremic metabolic alkalosis. Furthermore, the plasma chloride concentration tends to be significantly higher in *WNK1*^+*/FHHT*^:*SPAK*^*243A/243A*^ than in *SPAK*^*243A/243A*^ mice. The same result was obtained for NCC abundance and phosphorylation, which are lower than in control mice but higher than in *SPAK*^*243A/243A*^ mice. The inhibition of SPAK in *WNK1*^+*/FHHt*^ mice therefore results in an intermediate phenotype, between control and *SPAK*^*243A/243A*^ mice.

These results differ from what has been observed in mouse models carrying both a missense FHHt mutation in WNK4 and an inactivating mutation of SPAK (*Wnk4*^*D561A/*+^:*SPAK*^*243A/243A*^ or *Wnk4*^*D561A/*+^:*SPAK*^−/−^ mice)^15,16^. Indeed, the inhibition of SPAK in *Wnk4*^*D561A/*+^ mice restored blood pressure, potassium and acid-base balance as well as NCC abundance and phosphorylation to control levels. Taken together, these observations suggest that, even though they both can activate SPAK and OSR1 *in vitro*, L-WNK1 and WNK4 have different functions in the distal nephron, more particularly in the DCT. This is supported by the fact that NCC abundance and phosphorylation are drastically reduced in mice carrying a null mutation of *WNK4* (*WNK4*^−/−^)^28^. Therefore, the absence of WNK4 cannot be compensated for by L-WNK1. This could be due to the low level of physiological expression of L-WNK1 in the DCT^29^ since an overexpression of L-WNK1 in this segment can compensate for and even induce hyperkalemic and acidotic hypertension in the absence of WNK4^30^. One could therefore wonder if L-WNK1 plays a physiological role in the DCT. It is interesting to note that, whereas KS-WNK1 and WNK4 transcription in the mouse kidney is modulated by changes in Na^+^ or K^+^ intake and aldosterone, it is not the case for L-WNK1^31^. Recently, Roy *et al*. reported that L-WNK1 protein abundance is increased by aldosterone through the inhibition of its ubiquitination by Nedd4-2^32^. The precise characterisation of the roles played by L-WNK1 in the distal nephron is complicated by the fact that L-WNK1 knock-out impairs cardiovascular development and thus results in embryonic lethality^33^.

We also show for the first time that WNK1 cellular localisation is modified in the DCT of *WNK1*^+*/FHHt*^ and *WNK1*^+*/FHHT*^:*SPAK*^*243A/243A*^ mice. While it is located in cytoplasmic punctate structures in control mice, the WNK1 signal is found near the apical membrane in the mutant mice (Fig. 6). The antibody we used recognized both L-WNK1 and KS-WNK1. Boyd-Shiwarski and collaborators showed that, *in vitro*, transfected KS-WNK1 localises to cytoplasmic puncta, similar to those observed *in vivo* in DCT cells, while transfected L-WNK1 was distributed diffusively throughout the cytoplasm^34^. Interestingly, both isoforms were found in the puncta when cotransfected. The deletion of *WNK1* first intron, which corresponds to the FHHt mutation carried by the two mouse models, stimulates L-WNK1 transcription^18^. Taken together, these results suggest that, upon overexpression and probably activation, L-WNK1 translocates from the puncta to the apical membrane of DCT cells. Again, this result is different from what has been described for WNK4. Indeed, Terker and collaborators showed that WNK4 remains located in cytoplasmic punctate structures in the DCT of mice subjected to a potassium depletion, a situation in which WNK4 is overexpressed and activated by phosphorylation^23^. Whether the WNK4-positive puncta also contain WNK1 isoforms or whether there are two different structures remains to be defined.

The fact that NCC is still phosphorylated in *WNK1*^+*/FHHT*^:*SPAK*^*243A/243A*^ mice at a higher level than in *SPAK*^*243A/243A*^ mice suggest that NCC can be phosphorylated by another WNK1-activated kinase *in vivo*. The obvious candidate is OSR1, the phosphorylation of which tends to increase in *WNK1*^+*/FHHT*^:*SPAK*^*243A/243A*^ mice (α. 4). Grimm *et al*. showed that OSR1 is retained in cytoplasmic punctate structures in DCT cells in the absence of SPAK^26^, where it would not be able to phosphorylate NCC. The authors suggested that an interaction between SPAK and OSR1 could be necessary for the proper localisation of OSR1 near the apical membrane of DCT cells. This hypothesis is supported by the fact that OSR1 is still located near the apical membrane of *SPAK*^*243A/243A*^ DCTs (Fig. 5). Indeed, the SPAK protein is still present in these mice, the threonine to alanine mutation only precluding the activation by WNKs (Fig. 4 and^11^). Tsutsumi *et al*. showed that SPAK can translocate from the cytoplasm to the cytoskeleton, where it associates with F-actin, upon stress such as an incubation in a hypertonic solution^35^. It would be interesting to determine if the phosphorylation of the S-motif of SPAK is involved in the regulation of the intracellular localisation of the kinase. Indeed, if it is known that this residue is phosphorylated by the WNKs and that it is not required for the activation of SPAK^8^, its precise role remains to be characterized. Taken together, these observations suggest that OSR1 could be activated by L-WNK1 and partially compensate for the absence of SPAK in the setting of FHHt. However, at least a third protein could also phosphorylate NCC *in vivo* since the cotransporter is still phosphorylated and activated by potassium depletion, albeit at a very low level, in a mouse model carrying an inactivation of both SPAK and OSR1^23^. A potential candidate is the calcium-binding protein Cab39, which promotes the binding of WNK4 to NKCC1 and thus enables the activation of the cotransporter in a SPAK-independent manner^22^. Whether or not Cab39 is involved in the activation of NCC in *WNK1*^+*/FHHT*^:*SPAK*^*243A/243A*^ mice remains to be determined as we did not observe a change in Cab39 in the different mouse models.

In conclusion, the present study shows that SPAK activation is required downstream of L-WNK1 to stimulate NCC and provokes FHHt. It also suggests that L-WNK1 does not play the same role than WNK4 in physiological conditions, even though both kinases can activate SPAK in a similar manner *in vitro*. The physiological role played by L-WNK1 in the DCT, and more particularly the conditions in which it may be essential, remain to be defined.

## Material and Methods

### Generation of mice

*SPAK*^*243A/243A*^ and *WNK1*^+*/FHHt*^ mice (C57BL/6 J background) were described previously^11,18^. *SPAK*^*243A/243A*^ mice were first crossed with *WNK1*^+*/FHHt*^ to obtain *WNK1*^+*/FHHt*^:*SPAK*^+*/243A*^. Double heterozygous mice were then crossed to produce *WNK1*^+/+^:*SPAK*^+/+^ (controls), *WNK1*^+*/FHHt*^:*SPAK*^+*/*+^ (*WNK1*^+*/FHHt*^), *WNK1*^+*/FHHt*^:*SPAK*^*243/243A*^ and *SPAK*^*243/243A*^ mice. All studies were conducted on 3- to 5-month-old male mice and performed in accordance with the European Communities Council Directive. The project has been approved by the French Ministry of Research (#02650.02).

### Blood analysis

Blood was collected by retro-orbital puncture on anesthetized mice. Blood gas and electrolytes were analysed on an ABL80Flex analyser (Radiometer).

### Telemetry

Experiments were performed on 5 control and 5 *WNK1*^+*/FHHt*^:*SPAK*^*243A*:*243A*^ male mice. The catheter of the BP telemeter (model TA11PA-C10; Data Sciences International) was inserted into the right femoral artery. The telemetric transmitter probe was positioned subcutaneously on the flank. To reduce any infection and pain, the mice received one dose (20 mg/kg ip) of amoxicillin (Clamoxyl; SmithKlineBeecham Laboratories, Nanterre, France) and one dose (5 mg/kg ip) of ketoprofen (Profenid; Aventis, Paris, France). After the mice had recovered from the anesthesia in a warm (36 °C) box, they were housed in individual cages placed on top of the telemetric receivers in a light-dark cycled recording room (7:00–19:00). After a 1-wk recovery period, recordings of cardiovascular parameters and locomotor activity were obtained continuously during 24 h in the freely moving mice in their home cages every 15 minutes for 60 seconds. Each recording was visualized to select one segment without erratic fluctuations of enough duration (51.2 s) every 15 min (4 segments per hour) for the 24 hours i.e. 96 segments for each animal per day. The values for each recording segment were averaged over a 12-hour period (7:00–19:00 for day-time and 19:00–7:00 for night-time. Mice were fed standard A04 mouse chow (UAR, Epinay-sur-Orge, France) with water ad libitum.

### Immunoblot analysis

Renal cortex samples were homogenized in a cold extraction buffer containing 0.25 M sucrose, 20 mM tris-Hepes pH 7.4, proteases and phosphatase inhibitors (Complete and PhosSTOP tablets; Roche Diagnostics). The homogenates were then subjected to a first centrifugation (4,000 g for 15 min) to obtain post-nuclear fractions. The supernatant was centrifuged at 17,000 g for 20 min: the resulting pellet corresponds to the plasma membranes-enriched fraction. These fractions were then submitted to SDS/PAGE electrophoresis, and immunoblotting was performed as described^36^. The following antibodies were used: NCC (gift from D. Ellison), ENaC γ-subunit (gift from J. Loffing), pendrin (gift from S.Wall), NKCC2 phospho-Thr96-T101 (gift from K. Mutig), Cab39 (Cell signalling Technology^®^), NCC phospho-Thr55, SPAK, OSR1, SPAK/OSR1 phospho-S motif (S383/325) and NKCC2. The last five antibodies were obtained from the Division of Signal Transduction Therapy of the University of Dundee. As cropped images of the immunoblots are presented in the Figures, the full-length images are provided in the Supplementary Figures S3 to S5.

For all experiments, control gels were run prior to western blotting and were stained with Coomassie. An example of such a gel is provided in Supplementary Fig. S6. Several representative bands were then quantified by densitometry to assure equality of loading between samples^37^.

Quantification of the band(s) corresponding to the protein of interest was performed by densitometry using the Image J software. Densitometric values were normalized to the mean of the control group that was defined as 100%, and results were expressed as mean ± s.e.m.

### Immunofluorescence on frozen kidney sections

Kidneys were fixed by perfusion in the aorta with 10% formol - pH 7.0 (Microm-Microtech, France). Dissected kidneys were washed in cold PBS and frozen in isopentane cooled with liquid nitrogen. 4 µm thick cryosections were blocked with PBS-0.02% BSA and then incubated overnight at 4 °C with the primary antibodies [sheep anti-OSR1 and sheep anti-NCC (Division of Signal Transduction Therapy of the University of Dundee), rabbit anti-NCC (Millipore), and rabbit anti-WNK1 (Bethyl)]. After three 5-min washes with PBS, the sections were incubated with the two fluorophore-conjugated secondary antibodies [AlexaFluor™ 488 Donkey-anti Sheep, AlexaFluor™ 546 Goat-anti Rabbit (Invitrogen™, ThermoFisher Scientific)] for two hours at room temperature and then washed with PBS. Sections were mounted with the Mountant Permafluor medium (ThermoFisher Scientific). Representative images were acquired with an inverted Olympus IX83 microscope.

Quantification of the OSR1 signal was performed using the Image J software. Regions of interest (ROI) were set on individual DCTs as lines across the height of a single epithelial cell, avoiding the nucleus. Five ROIs, in average, were traced for each DCT and at least 7 tubules were analysed per animal. The intensity of each pixel of the ROI was measured. The background, measured of each section, was measured and subtracted. We then calculated two means: (1) the mean of the intensities along the whole ROI and (2) the mean of the intensities of the pixels located in the first quarter of the ROI, near the apical membrane (Supplementary Figure S7). The ratio between these two values allowed us to evaluate the fraction of OSR1 protein located near the apical membrane.

### Statistical analysis

When analysing two groups of mice, we used an unpaired student t-test. An one-way ANOVA followed by Sidak’s multiple comparisons test was used to analyse more than two groups with n ≥5 while a Kruskal-Wallis followed by a Mann-Whitney test was used to analyse more than two groups with n <5. Data are given as mean ± s.e.m. A difference between groups was considered significant when *P* < 0.05.

### Materials and Data availability

All data generated or analysed during this study are included in this published article (and its Supplementary Information files). All the materials and protocols, except those generated by other laboratories as stated above, are available from the corresponding author.

## Electronic supplementary material

Supplementary Figures
