# Supplementary material for: Consequences of SPAK inactivation on Hyperkalemic Hypertension caused by WNK1 mutations: evidence for differential roles of WNK1 and WNK4
Source: Sci Rep. 2018 Feb 19;8:3249. doi: 10.1038/s41598-018-21405-x (PMC5818654; doi:10.1038/s41598-018-21405-x)
Supplement: Supplementary file 1 — Supplementary Figures [file 41598_2018_21405_MOESM1_ESM.pdf]

SUPPLEMENTARY FIGURES ONLINE

Consequences of SPAK inactivation on Hyperkalemic Hypertension caused by *WNK1* mutations: evidence for differential roles of WNK1 and WNK4.

Chloé Rafael, Christelle Soukaseum, Véronique Baudrie, Perrine Frère
and Juliette Hadchouel

Supplementary Figure S1

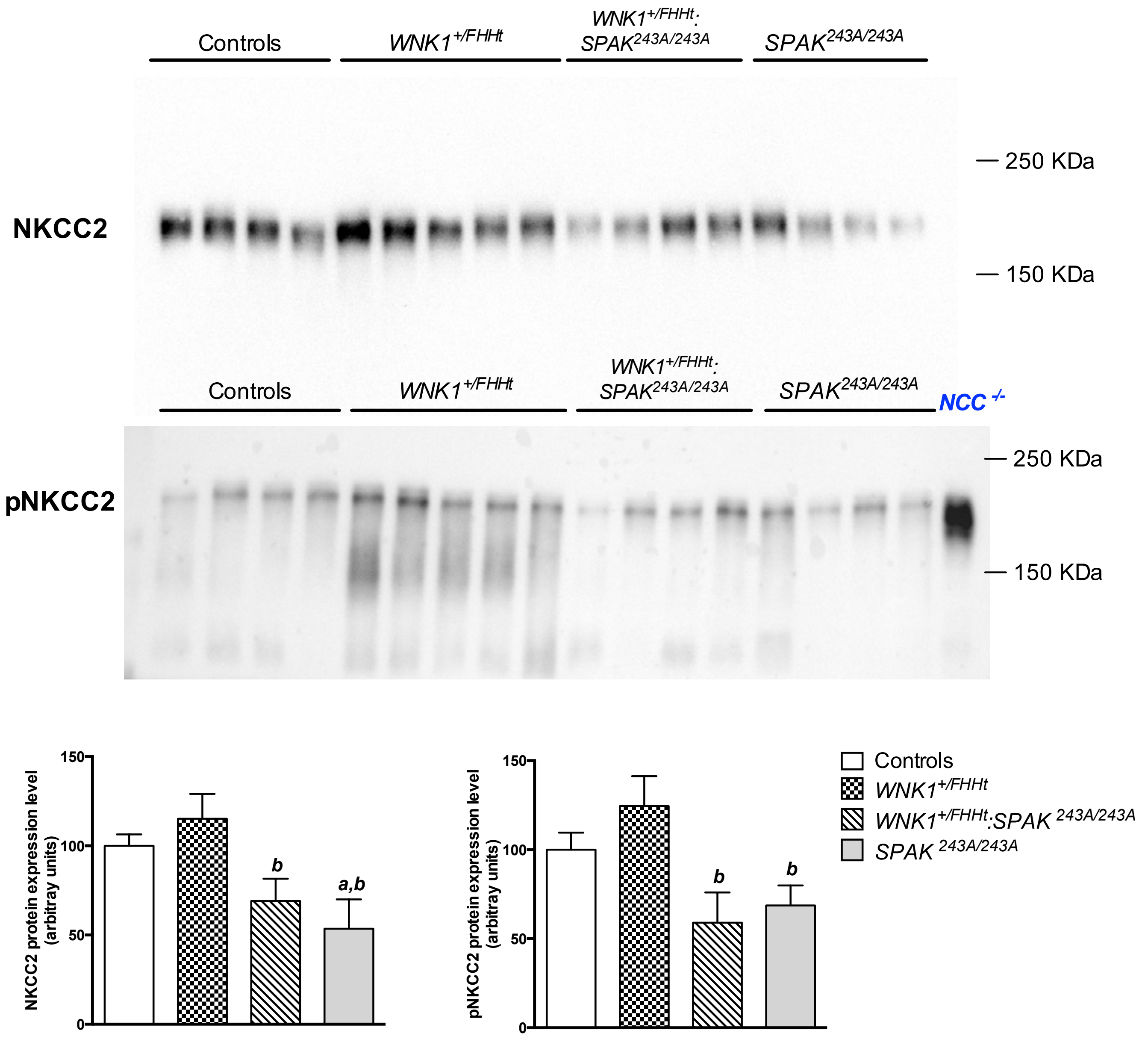

Supplementary Figure S1. The expression and phosphorylation of NKCC2 are decreased in *$WNK1^{+/FHHt}$:$SPAK^{243A/243A}$* mice.

Upper panel: Representative immunoblots of NKCC2 and phosphorylated NKCC2 performed on the membrane-enriched fractions of the renal cortex of mice of each genotype. As pNKCC2 antibody is also able to recognize pNCC, we loaded a protein sample extracted from a *$NCC^{-/-}$* mouse kidney (in blue, last lane of the pNKCC2 blot) to identify the band corresponding specifically to pNKCC2.

Lower panel: Densitometric analysis. NKCC2 abundance and phosphorylation are decreased in *$WNK1^{+/FHHt}$:$SPAK^{243A/243A}$* and *$SPAK^{243A/243A}$* mice. In contrast to NCC, there is no difference between these two groups. Number of animals: 4 control, 5 *$WNK1^{+/FHHt}$*, 4 *$WNK1^{+/FHHt}$:$SPAK^{243A:243A}$* and 4 *$SPAK^{243A:243A}$* male mice. Values are means ± s.e.m. ^{a} p<0.05 vs. controls. ^{b} p<0.05 vs. *$WNK1^{+/FHHt}$* mices (Kruskal-Wallis one-way analysis of variance, followed by Mann-Whitney test). Immunoblot images are full-lenght images.

Supplementary Figure S2

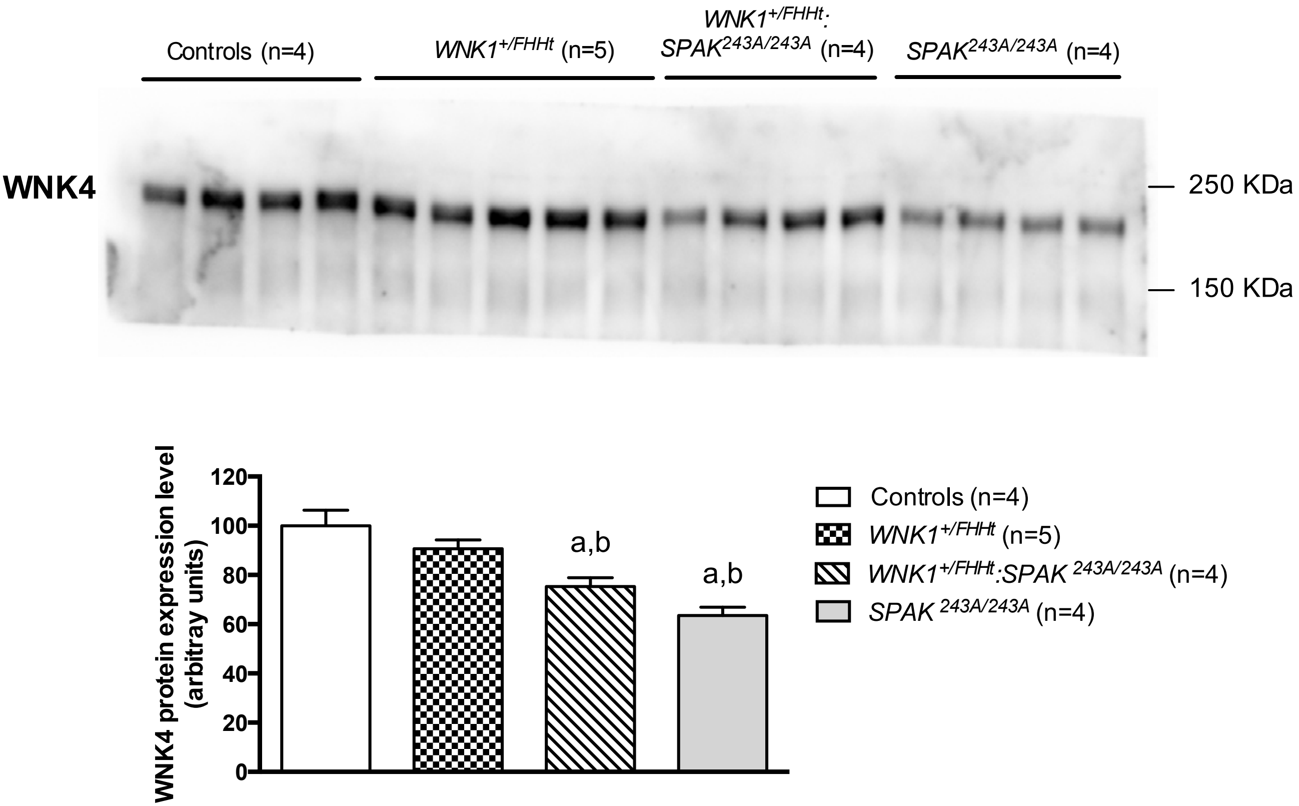

Supplementary Figure S2. WNK4 expression is decreased in *$WNK1^{+/FHHt}$:$SPAK^{243A:243A}$* mice.
Upper panel: Representative immunoblot of WNK4 performed on protein extracted from the renal cortex of mice of each genotype. **Lower panel:** Densitometric analysis. WNK4 abundance is decreased in *$WNK1^{+/FHHt}$:$SPAK^{243A/243A}$* and *$SPAK^{243A/243A}$* mice compared to control mice. Number of animals: 4 control, 5 *$WNK1^{+/FHHt}$*, 4 *$WNK1^{+/FHHt}$:$SPAK^{243A:243A}$* and 4 *$SPAK^{243A:243A}$* male mice. Values are means ± s.e.m. ^{a} $p<0.05$ vs. controls. ^{b} $p<0.05$ vs. *$WNK1^{+/FHHt}$* mice. The immunoblot image is a full-lenght image.

Supplementary Figure S3

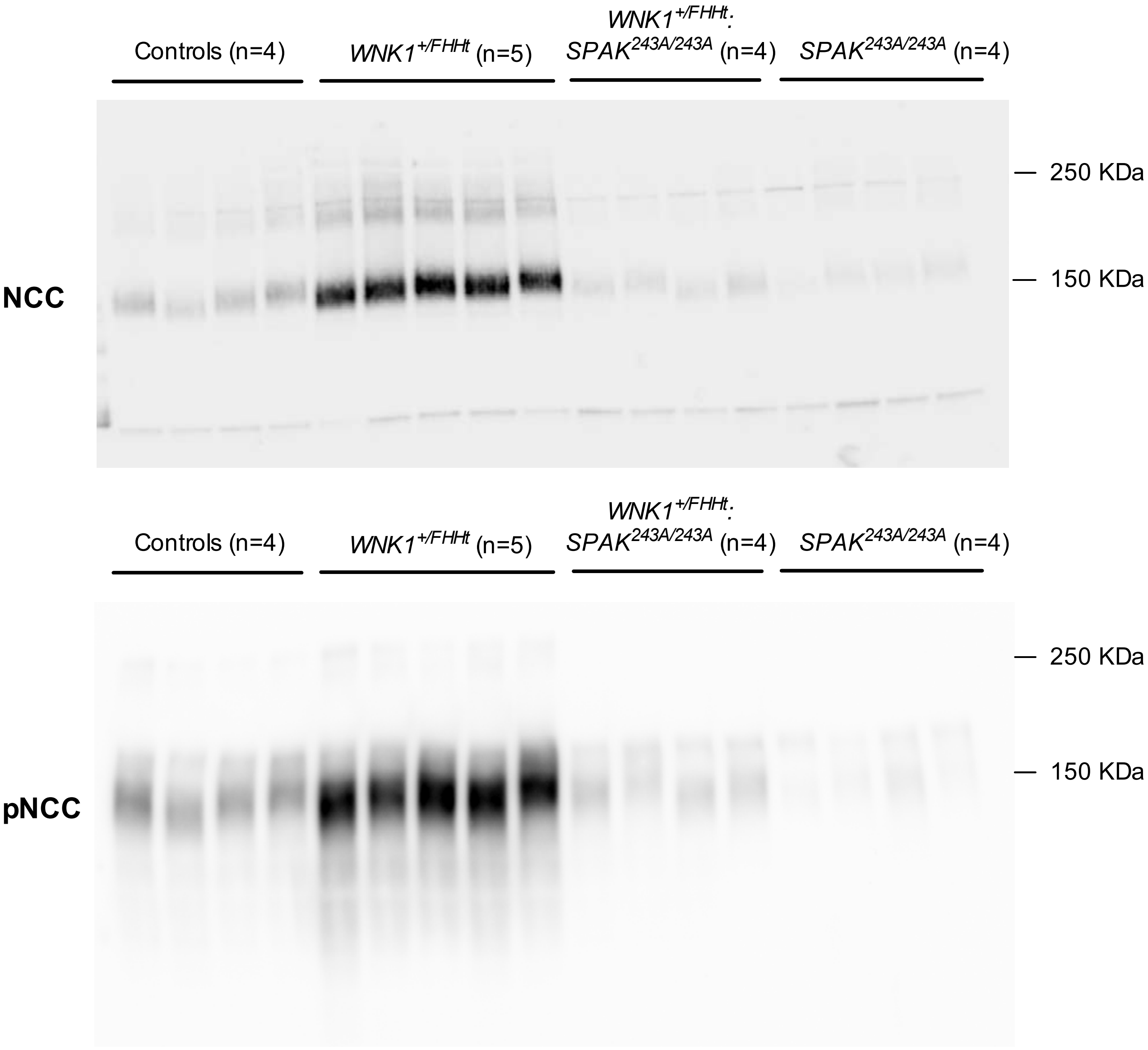

Supplementary Figure S3. Full-lenght iamges of the NCC and pNCC immunoblots, corresponding to Figure 2.

Supplementary Figure S4

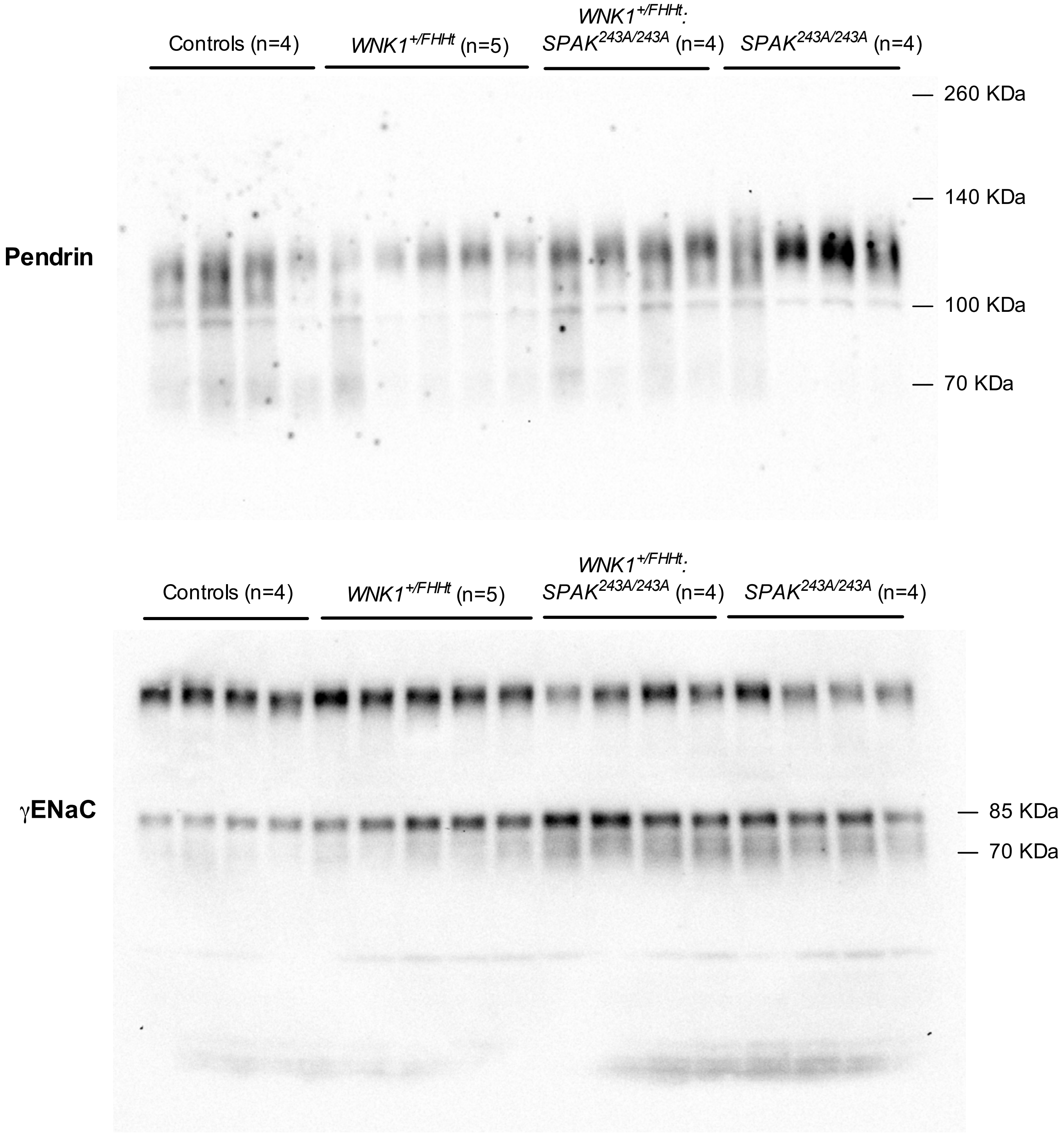

Supplementary Figure S4. Full-lenght iamges of the pendrin and γENAC immunoblot, corresponding to Figure 3.

Supplementary Figure S5

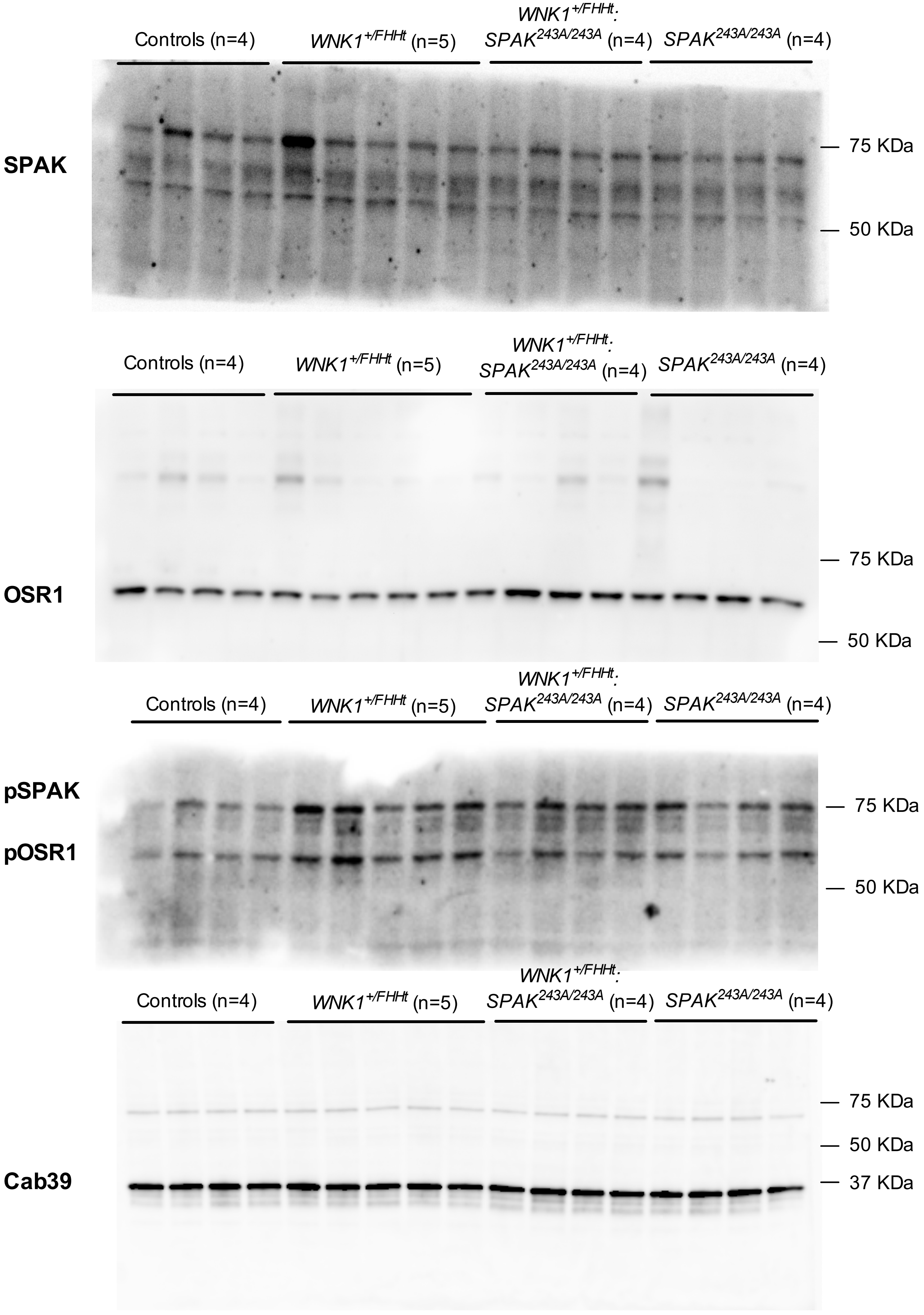

Supplementary Figure S5. Total SPAK, total OSR1, pSPAK/pOSR1 (S-motif) and Cab39 full-lenght immunoblot images.

Supplementary Figure S6

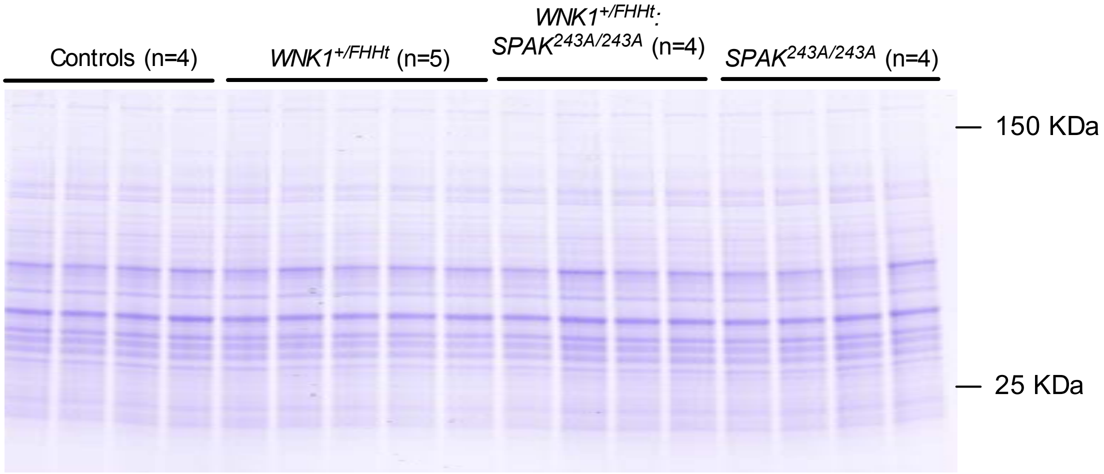

Supplementary Figure S6. Representative Coomassie gel staining. Preliminary 10% SDS-PAGE gels were run and stained with Coomassie blue to confirm equality of loading in each lane.

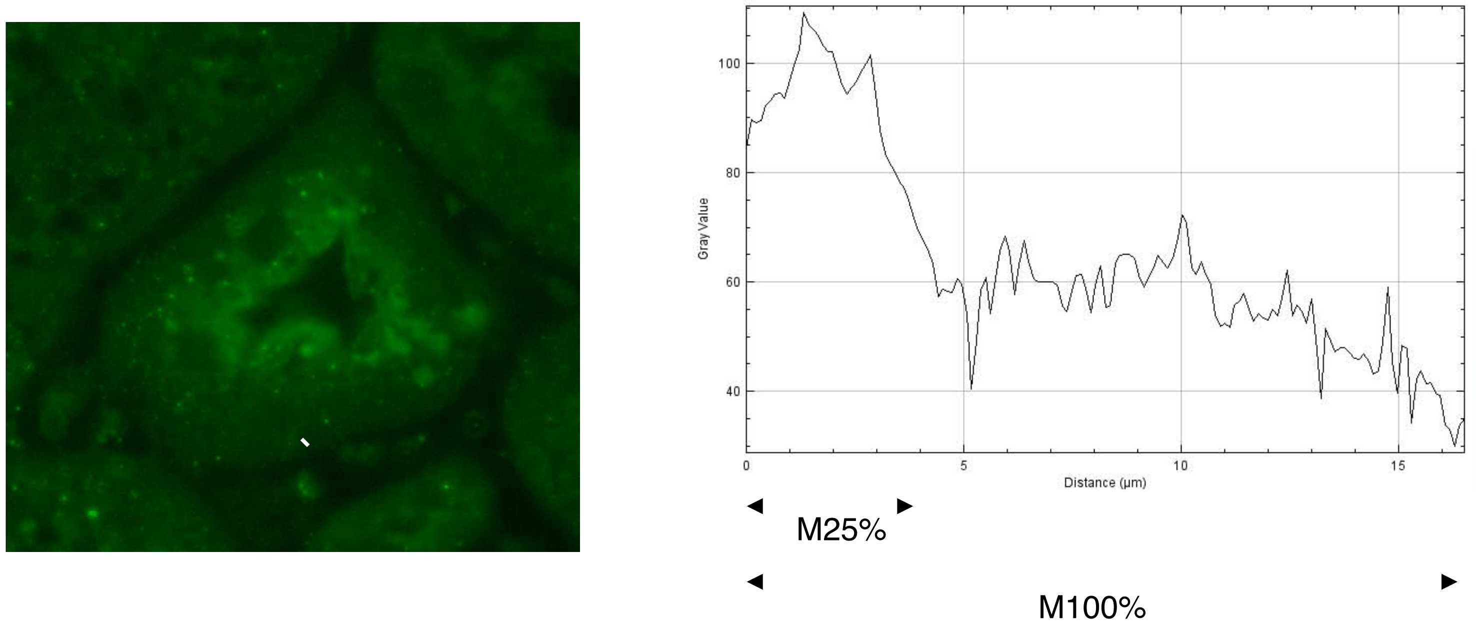

L = length of the ROI

M25% = Mean intensity of pixels from 0 (apical membrane) to L/4

M100% = Mean intensity of all pixels

Apical OSR1 = M25% / M100%

Supplementary Figure S7. Schematic description of the method used to quantify OSR1 apical staining. A detailed description is provided in the Material and Methods section.
